# Multiple Skin Adnexal Tumours with Possible Syndromic Association

**DOI:** 10.7759/cureus.102352

**Published:** 2026-01-26

**Authors:** Janavi Sridhar, Archana Balasubramanian, Leena Joseph, Deyonna Fernandez, Ramesh B A

**Affiliations:** 1 School of Medicine, Sri Ramachandra Institute of Higher Education and Research, Chennai, IND; 2 Pathology, Sri Ramachandra Institute of Higher Education and Research, Chennai, IND; 3 Plastic and Reconstructive Surgery, Sri Ramachandra Institute of Higher Education and Research, Chennai, IND

**Keywords:** brooke-spiegler syndrome, cyld cutaneous syndrome, cyld gene, skin adnexal tumors, spiradenoma

## Abstract

Brooke-Spiegler syndrome (BSS), multiple familial trichoepithelioma (MFT1) and familial cylindromatosis (FC) are autosomal dominant tumor syndromes that predispose individuals to multiple benign and malignant tumors, morphologically related to the adnexal structures of the skin. As allelic conditions caused by mutations in the *CYLD* gene, they are considered variants of a spectrum termed CYLD cutaneous syndrome (CCS). Patients commonly present with multiple adnexal tumors, such as cylindromas, trichoepitheliomas and spiradenomas, gradually increasing in size and number. Here, we report the case of a woman in her fifties who presented with an infected wound and multiple enlarging skin tumors over the face, scalp and upper back. Histopathological studies confirmed multiple skin adnexal tumors with features of eccrine spiradenoma, foci of trichoepithelioma and cylindroma areas. The patient underwent excision of the lesions with skin grafts and, given the possibility of syndromic association, was advised *CYLD *gene testing.

## Introduction

Cutaneous adnexal tumors are neoplasms that are morphologically linked to adnexal structures of the skin. They can be benign or malignant and occur sporadically or as part of tumor syndromes, such as CYLD cutaneous syndrome (CCS), a spectrum comprising multiple familial trichoepithelioma 1 (MFT1), Brooke-Spiegler syndrome (BSS), and familial cylindromatosis (FC) [1].

CCS is underreported worldwide, with an estimated prevalence in the United Kingdom of around 1:100,000 [2]. Geographical clusters of cases have been reported, often with multiple generations of affected family members [3]. While individual cases of CCS have been documented from Asia and the Indian subcontinent, the exact prevalence of the disease remains unknown [4-6]. Earlier studies reported a higher incidence among women; however, broader pedigrees have revealed equal penetrance among both sexes, with increased expressivity in females [1].

CCS exhibits variable expressivity, even within families, and typically presents in late childhood or early adulthood with spiradenomas, cylindromas, trichoepitheliomas, and other skin appendage tumors [7]. These tumors commonly occur due to inherited germline mutations in the *CYLD *tumor suppressor gene, but can also occur in patients without a family history due to de novo mutations; post-zygotic mutations can lead to mosaic presentations [8]. Salivary gland tumors and pulmonary cylindromas are less common manifestations, and malignant transformation, though documented, is rare [1].

In patients presenting with multiple skin adnexal tumors, early recognition and clinical and histopathological distinction from sporadic differential diagnoses can aid in prompt confirmatory genetic testing and the establishment of close follow-up to monitor recurrence and detect malignant lesions. Early surgical management is also crucial to reduce tumor burden and associated disfigurement [7]. Additionally, patients diagnosed with CCS are advised to undergo periodic dermatological evaluation to monitor new tumor development as well as signs of malignant transformation [1].

Our patient was in her teens when her tumors initially appeared, within the age range expected for patients with CCS. Multiple adnexal tumor patterns in the histology also pointed toward a syndromic link. Her case highlights the diagnostic challenges associated with such a clinical presentation and the potentially severe tumor burden of CCS.

## Case presentation

History and examination

A woman in her late fifties presented with complaints of multiple painless tumors over the face, scalp, and upper back, gradually enlarging over the past five years, and an infected ulcerated wound behind the right ear for the past week. The tumors initially appeared when she was in her teens. The patient and her family were offered the option of ablative treatment, but chose not to undergo the same due to concerns about its cosmetic side effects. The tumors remained small until five years ago, when the patient began to notice a gradual increase in size. The patient developed pain and a foul smell from the site of the infected lesion one week prior to presentation, but no pain was associated with the other tumors on the face, scalp, and upper back. She was referred to the hospital for further treatment by a dermatologist with an initial clinical diagnosis of neurofibromatosis. There was no evidence of systemic involvement. The patient had no children and no family history of similar complaints. She had no known comorbidities or relevant surgical history apart from cataract operations on both eyes two years prior.

**Figure 1:**
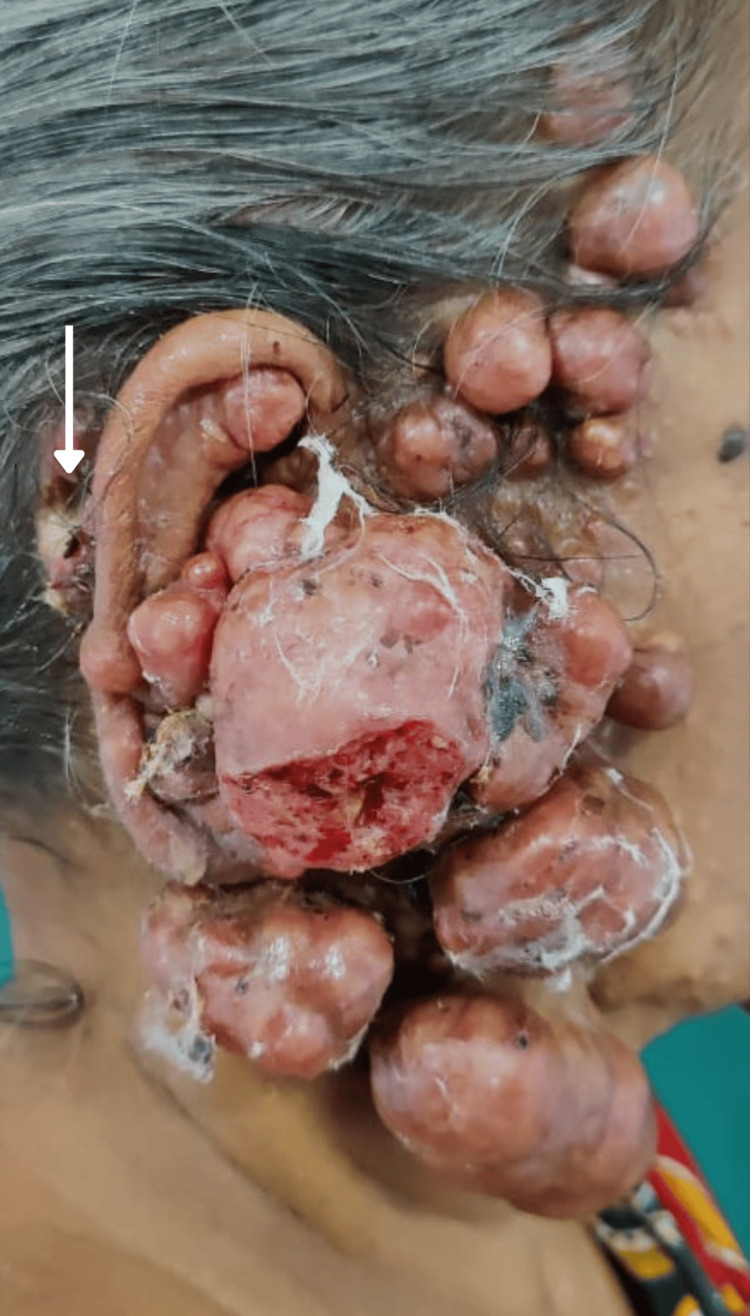
Right lateral view of facial lesions. Several nodular swellings of varying sizes in the right ear, preauricular region, and scalp, and an infected ulcer with seropurulent discharge in the postauricular region (white arrow).

**Figure 2:**
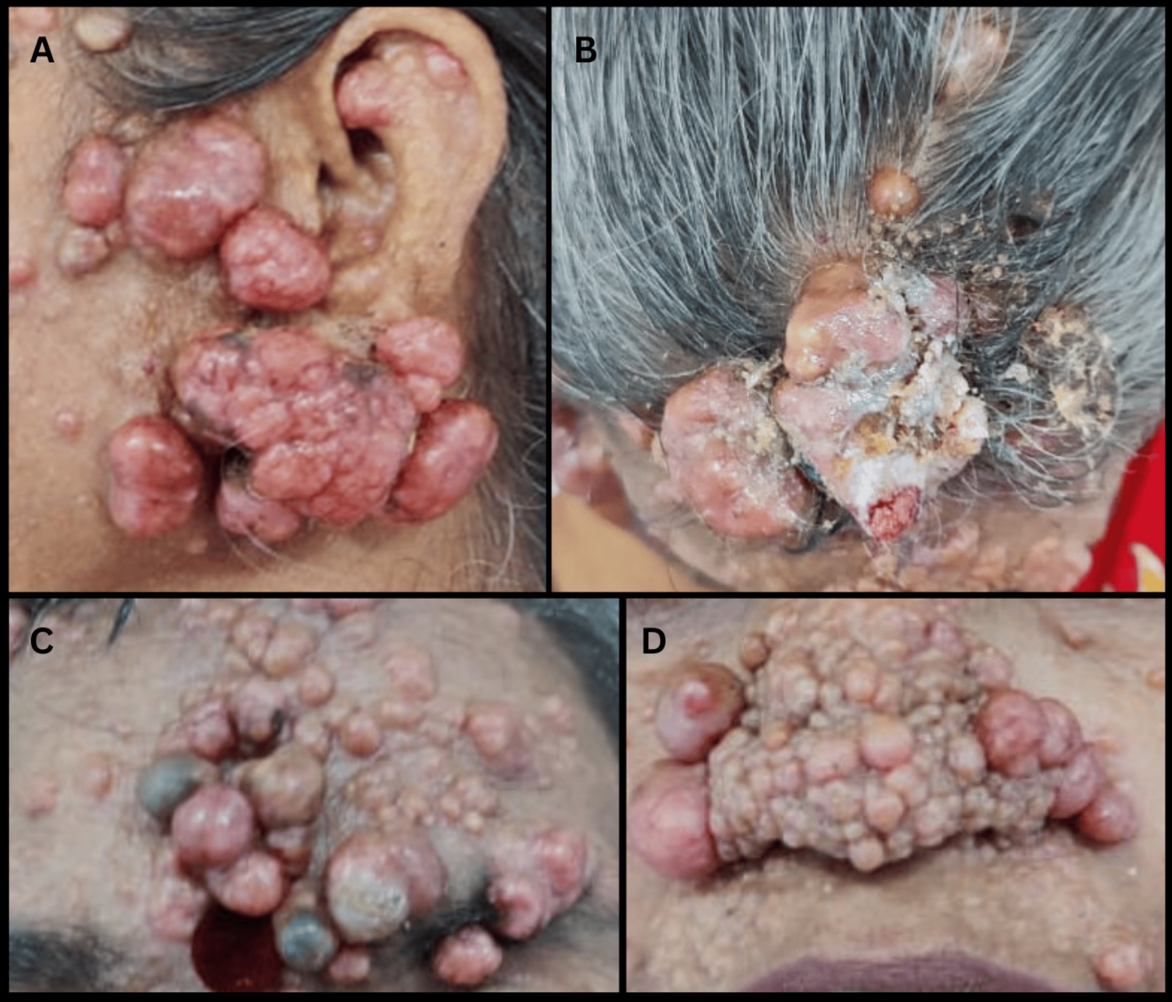
Multiple lesions over the face and scalp. A: left lateral view of swellings in the left preauricular region; B: axial view of fungating scalp lesions; C: lesions on the forehead involving the area extending from the hairline superiorly to the left supra-orbital ridge inferiorly; D: multiple swellings over the nose and involving the nasolabial folds.

The patient was moderately built, and her vital signs, general and systemic examinations were normal. On local examination, numerous pinkish and skin-colored nodular, pedunculated and sessile neoplasms were visible over the face, particularly involving the right preauricular and postauricular areas (Figure 1), the left preauricular area, forehead, scalp, nose, nasolabial folds, and upper back (Figure 2). The lesions had an asymmetrical distribution and varied in size, the largest measuring 6.5x5.5x5.0cm. They were smooth, firm, and non-tender on palpation. Examination of the right postauricular region revealed an infected ulcer measuring 1.0x1.0 cm with irregular margins, with hematoma, maggots, and necrotic slough on the ulcer floor (Figure 1).

Investigations

On admission, hematological investigations revealed microcytic hypochromic anemia (Haemoglobin: 10.2 g/dL) with normal white blood cell counts and no electrolyte disturbances or abnormalities in renal function (Table 1). Investigations for surgical fitness, including chest X-ray and electrocardiography, revealed no abnormalities. The patient subsequently underwent wound debridement and excision biopsy of lesions in the scalp, preauricular, and postauricular regions, with samples sent for histopathological analysis.

**Table 1:** Laboratory investigations at admission. g/dL: grams per deciliter; cells/µL: cells per microliter; fL: femtoliters; pg: picograms, mmol/L: millimoles per liter; mg/dl: milligrams per deciliter.

Parameters	Result	Units	Reference Range
Hemoglobin (Hb)	10.2	g/dL	12.0-15.0
Total white blood cell count (TC)	8720	cells/µL	4000-11000
Neutrophils	66.5	%	45-70
Immature granulocytes	0.6	%	<1.0
Lymphocytes	23.3	%	25-40
Eosinophils	5.2	%	1 – 6
Monocytes	3.9	%	2 -10
Basophils	0.5	%	0- 1
Packed cell volume (PCV)	34.3	%	36-46
Mean corpuscular volume (MCV)	75.6	femtoliters (fL)	83-101
Mean corpuscular hemoglobin (MCH)	22.5	picograms (pg)	27-33
Mean corpuscular hemoglobin concentration (MCHC)	29.7	g/dL	31.5-34.5
Platelet count	3.64	10^5^/µL	1.5-4.5
Red blood cell count	4.54	10^6^/µL	3.8-4.8
Prothrombin time (PT)	11.3	seconds	11.02-14.60
PT (control value)	12.2	seconds	11.02-14.60
PT International normalised ratio (INR)	0.92		0.9-1.1
Partial thromboplastin time (PTT)	20.6	seconds	25.1-30.9
PTT (control)	25.4	seconds	25.1-30.9
Glycated hemoglobin (HbA1c)	6	%	Normal: <5.7; Prediabetes: 5.7-6.4; Diabetes: ≥6.5
Sodium	139	mmol/L	136-145
Potassium	4	mmol/L	3.5-5.1
Chloride	104	mmol/L	98-107
Bicarbonate	23	mmol/L	22-29
Blood urea nitrogen (BUN)	10	mg/dL	6.0-20.0
Creatinine	0.7	mg/dL	0.5-0.9

Histopathology

**Figure 3:**
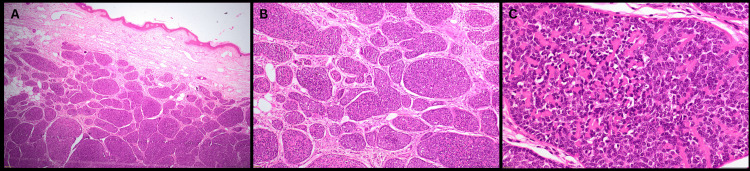
Histopathology images of excised neoplasms (Hematoxylin and Eosin stains used). A: H&E, 4x, section shows skin with dermal lesion composed of nests of tumor cells; B: H&E, 10x, section shows multiple nodules of closely packed basophilic tumor cells enclosing eosinophilic basement membrane-like material; C: H&E, 40x. High power view showing round to oval tumor cells with scant cytoplasm, fine chromatin, enclosing eosinophilic basement-membrane-like material. H&E: Hematoxylin and Eosin.

The excised specimens were gray-white and nodular on gross examination. No pathological changes were evident in the epidermis on microscopic examination. The dermis showed multiple nodules of closely packed basophilic tumor cells with focal areas resembling cylindromas in a jigsaw pattern, surrounded by an eosinophilic basement membrane material (Figure 3). Also present in the dermis were benign-appearing lesions with lymphocytic infiltration resembling eccrine spiradenoma, and foci of trichoepithelioma with dystrophic calcification were noted. Other observations included cholesterol clefts surrounded by foci of cystic degeneration and giant cell reaction, and a dense eosinophilic infiltrate in the dermis below the foci of epidermal erosion. Histology showed no evidence of atypia or necrosis (Figure 3). As immunohistochemistry is considered supportive but not required for diagnosis, it was not performed for this patient.

These microscopic findings suggested multiple skin adnexal tumors, with the combination of pathological types suggestive of a positive syndromic association such as Brooke-Spiegler syndrome. The patient was recommended *CYLD* gene testing to confirm the diagnosis, but opted not to undergo testing due to financial constraints.

Treatment

On admission, the patient was diagnosed with an active infection, with purulent, foul-smelling discharge and maggot infestation on the ulcerated nodules. She was treated initially with serial debridements, intravenous third-generation cephalosporins, and topical antibiotics (mupirocin). The patient underwent an excision biopsy of lesions in the preauricular, postauricular, and scalp regions under antibiotic cover six days later. Analysis of specimens sent for histopathological analysis confirmed the diagnosis of multiple adnexal tumors. The patient underwent excision of the remaining lesions, under intravenous antibiotic cover (cefazolin 1g), from the angle of the mandible, right preauricular region and postauricular region, right ear lobe antihelix, left preauricular and earlobe region, scalp, forehead, and nose.

**Figure 4:**
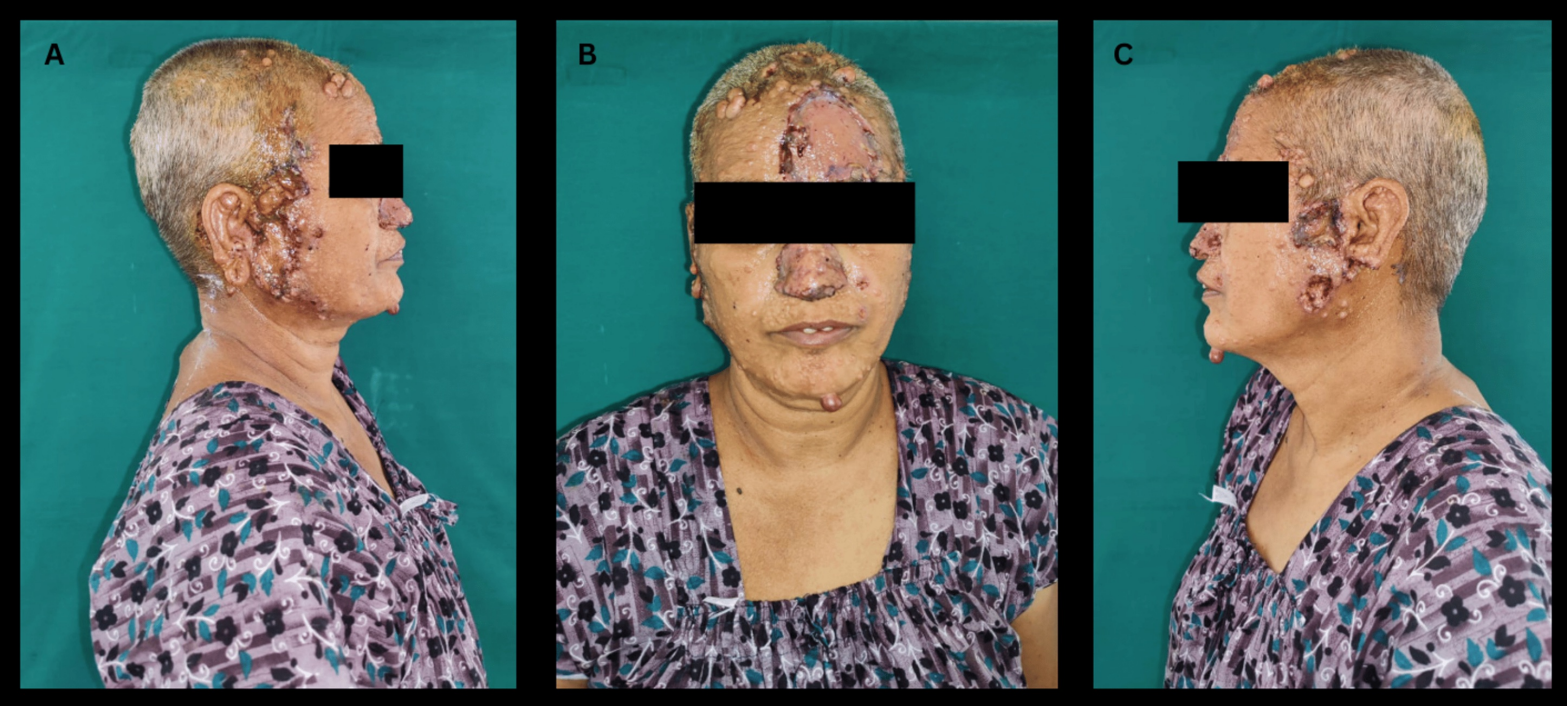
Postoperative images following excision of face and scalp lesions and skin cover. A: Postoperative image showing skin cover over the right pre-auricular area and nose with topical antibiotic application; B: Postoperative image showing skin cover over the forehead and nose with topical antibiotic application; C: Postoperative image showing skin cover over the left pre-auricular area with topical antibiotic application.

Based on their size, the raw areas were resurfaced with autologous split skin grafts or with primary sutures. Split skin grafts were applied over the nose, scalp, preauricular, and postauricular areas, and wound dressing was done with sterile gauze (Figure 4).

**Figure 5:**
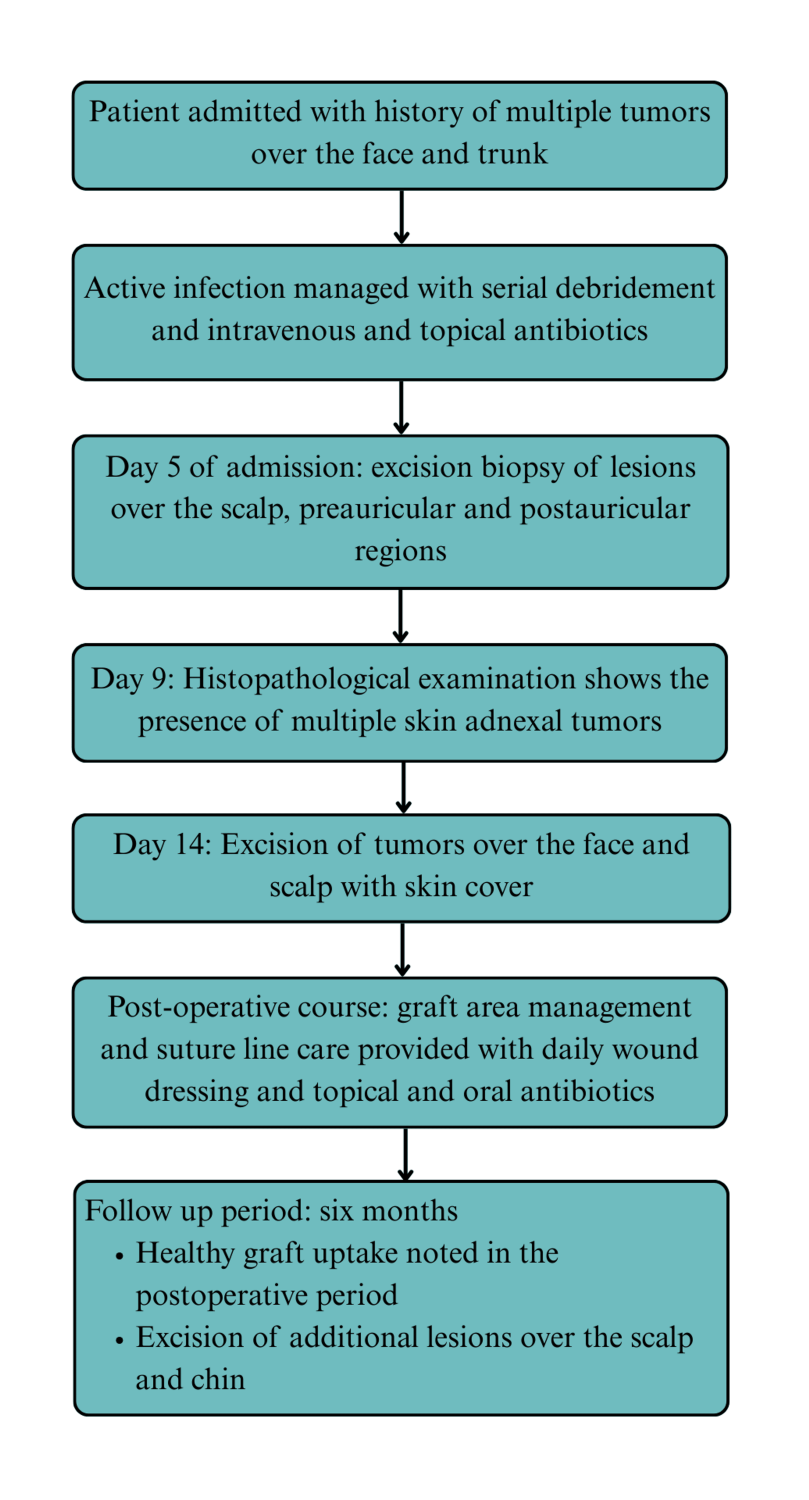
Flowchart illustrating the patient’s treatment course from initial presentation through postoperative management and follow-up.

The patient received intravenous crystalloids and antibiotics (ceftriaxone and sulbactam) in the postoperative period and before being shifted to the ward in stable condition. The graft areas and suture lines were maintained with daily wound dressing and topical antibiotic (mupirocin) application, along with ensuring strict head-end elevation, and the patient completed a one-week course of oral antibiotics (amoxicillin/clavulanate). After ensuring healthy graft uptake, the patient was discharged with instructions to continue applying mupirocin ointment over the wound areas and maintaining head-end elevation. She was prescribed an iron tonic fortified with folic acid to correct anemia, and educated on graft and donor site care. Over the next six months, the patient underwent excision of the remaining lesions and continued regular follow-up on an outpatient basis. The patient’s treatment course has been summarized below (Figure 5).

## Discussion

CYLD lysine 63 deubiquitinase (CYLD) is a protease encoded by the *CYLD* gene. It regulates critical cellular pathways and interactions through breaking ubiquitin chains bound to cellular proteins [8]. *CYLD* is located on chromosome 16q12.1 and is expressed in fetal and adult cells, particularly keratinocytes, oligodendrocytes, Langerhans cells, and immune cells. Several factors, including methylation of CpG islands and microRNA-mediated silencing, regulate the expression of *CYLD* [8].

The CYLD enzyme impacts multiple cell signaling pathways. It downregulates the NF-κB signaling pathway, which requires ubiquitin chains for activation, by cleaving targets TRAF2, TRAF6, TAK1, and NEMO [8]. It prevents the sequestration of β-catenin, causing a reduction in Wnt-signal transduction. Deubiquitination of TAK1 also regulates the MAPK signaling pathway, inhibiting p38-mediated gene expression. In the TGF-β pathway, the inhibition of SMAD2/3 phosphorylation is prevented by SMAD7 deubiquitination by CYLD [8]. The role of CYLDas a regulator in these pathways is evident from the increased risk of malignancies in individuals with weakened or truncated forms of the enzyme. In addition to CCS, pathogenic *CYLD* mutations are implicated in myeloma, melanoma, hepatocellular carcinoma, hematological malignancies, and several other sporadic cancers [8]. CYLD is also integral to autophagic and pro-apoptotic pathways, as well as cell cycle regulation through BCL-3, with gain-of-function mutations implicated in neurodegenerative diseases including amyotrophic lateral sclerosis, and autosomal-dominant frontotemporal dementia [9].

Patients with CCS are susceptible to tumor formation due to germline loss-of-function mutations in the *CYLD* gene. Most pathological variants have a truncated protein, usually due to frameshift mutations, affecting the ubiquitin-specific protease catalytic domain. Individuals with missense mutations have reduced deubiquitinase activity, and rarer variants arise from larger deletions and rearrangements. A second, loss of heterozygosity mutation (LOH), contributes to tumor formation. Mutations in both alleles result in loss of the deubiquitination function of CYLD, leaving skin appendages and other tissues vulnerable to uncontrolled proliferation and tumor formation [8]. CCS can present sporadically and in an inherited fashion, and affected individuals develop multiple skin adnexal tumors in adolescence or early adulthood [1]. Additional mutations are typically present in CCS, with TP53 mutations frequently present in patients with malignant transformation [10]. Co-localization of trichoepithelioma, cylindroma, and spiradenoma, each with specific identifying features, is characteristic of BSS and CCS [1].

While syndromic association can be suspected based on the clinical and histological profile of the patient, only genetic testing can confirm CCS, which involves assaying blood leukocyte samples for mRNA produced by the gene. In mosaic patterns, with a varied distribution of mutations between leukocytes and hair follicles, *CYLD* gene mutations may go undetected. Deep intronic mutations can also be missed, in which the gene encodes a non-functional protein but can still be detected as unremarkable in the assay [7]. The morbidity of CCS in affected individuals is often severe. The lesions can be numerous and disfiguring, and pain, bleeding, and ulceration are frequent complications. Many patients require multiple surgeries to remove the lesions [11].

At the time of her admission, the patient had been clinically diagnosed with neurofibromatosis in the outpatient department. The clinical features suggestive of this diagnosis were the age of onset of the face and scalp lesions and their distribution; multiple neoplasms pointed towards a syndrome-associated condition rather than a sporadic occurrence. However, the patient did not present with other features suggestive of Neurofibromatosis types 1 and 2, such as hyperpigmented macules or cafe-au-lait macules, pain or itching, Lisch nodules, and intertriginous freckles (NF1), or vestibulocochlear nerve involvement (NF2) [12]. The absence of spindle cells, divergent differentiation, and marbleized appearance upon histological examination, all features of peripheral nerve sheath tumors such as neurofibromatosis, also called for an alternate diagnosis [12].

The clinical presentation of multiple facial neoplasms has several differential diagnoses, including Birt-Hogg-Dube syndrome, tuberous sclerosis complex, Cowden syndrome, and nevoid basal cell carcinoma syndrome. In Birt-Hogg-Dube syndrome, patients typically present with fibrofolliculomas, small, whitish or yellowish dome-shaped opaque papules which begin in the nasal and paranasal areas before involving broader areas of the face and trunk [13]. Additional cutaneous lesions include trichodiscomas and acrochordons, and other distinguishing features include pulmonary cysts, which can manifest as spontaneous pneumothorax, renal cell carcinomas, and histological evidence of circumscribed fibrosis [13]. Tuberous sclerosis complex presents with hypopigmented macules, shagreen patches, ungual fibromas and angiofibromas, along with the involvement of the brain (TSC-associated neuropsychiatric disorder, seizures), kidney (renal angiomyolipomas), and other organs [14]. In addition to solid organ malignancies and cranial abnormalities, cutaneous manifestations of Cowden syndrome include trichilemmomas and acral and plantar keratoses [15]. Common presentations in nevoid basal cell carcinoma syndrome include basal cell carcinomas and characteristic jaw keratocysts, frontal bossing, macrocephaly and coarse facies [16]. The absence of systemic involvement, associated clinical features, and characteristic histological findings ruled out these diagnoses.

In this case, multiple painless, enlarging tumors over the face, scalp, and trunk, accompanied by the age of onset, histological pattern, and the absence of systemic involvement, pointed to a diagnosis of multiple adnexal tumors strongly suggestive of CCS. The patient opted not to undergo confirmatory genetic testing.

## Conclusions

CCS is a rare disease, and awareness of its presentation in ethnically diverse populations and features distinguishing it from similar conditions can aid in timely diagnosis and intervention. This case demonstrates the potential for severe tumor burden in CCS and its consequent cosmetic and psychological impact on affected individuals. While our patient’s clinical presentation of multiple painless enlarging neoplasms over the face and trunk, the age of onset of symptoms and histological pattern are strongly suggestive of CCS, the diagnosis remains presumptive without confirmatory genetic testing. Additionally, patients diagnosed with CCS are advised frequent dermatological follow-up to monitor new tumor development and recognize signs of malignant transformation. The lack of genetic testing, as well as the shorter period of follow up, were some of the limitations we encountered in this study.
